# Within-host evolution decreases virulence in an opportunistic bacterial pathogen

**DOI:** 10.1186/s12862-015-0447-5

**Published:** 2015-08-19

**Authors:** Lauri Mikonranta, Johanna Mappes, Jouni Laakso, Tarmo Ketola

**Affiliations:** Centre of Excellence in Biological Interactions, Department of Biological and Environmental Science, University of Jyväskylä, P.O. Box 35, 40014 Jyväskylä, Finland; Centre of Excellence in Biological Interactions, Department of Biological and Environmental Science, University of Helsinki, University of Helsinki, P.O. Box 65, 00014 Helsinki, Finland

## Abstract

**Background:**

Pathogens evolve in a close antagonistic relationship with their hosts. The conventional theory proposes that evolution of virulence is highly dependent on the efficiency of direct host-to-host transmission. Many opportunistic pathogens, however, are not strictly dependent on the hosts due to their ability to reproduce in the free-living environment. Therefore it is likely that conflicting selection pressures for growth and survival outside versus within the host, rather than transmission potential, shape the evolution of virulence in opportunists. We tested the role of within-host selection in evolution of virulence by letting a pathogen *Serratia marcescens* db11 sequentially infect *Drosophila melanogaster* hosts and then compared the virulence to strains that evolved only in the outside-host environment.

**Results:**

We found that the pathogen adapted to both *Drosophila melanogaster* host and novel outside-host environment, leading to rapid evolutionary changes in the bacterial life-history traits including motility, *in vitro* growth rate, biomass yield, and secretion of extracellular proteases. Most significantly, selection within the host led to decreased virulence without decreased bacterial load while the selection lines in the outside-host environment maintained the same level of virulence with ancestral bacteria.

**Conclusions:**

This experimental evidence supports the idea that increased virulence is not an inevitable consequence of within-host adaptation even when the epidemiological restrictions are removed. Evolution of attenuated virulence could occur because of immune evasion within the host. Alternatively, rapid fluctuation between outside-host and within-host environments, which is typical for the life cycle of opportunistic bacterial pathogens, could lead to trade-offs that lower pathogen virulence.

## Background

In order to reproduce and to transmit forward, pathogens must draw resources from their host and hence cause harm to them. The term virulence describes the level of this pathogen induced host damage [1]. The fitness of an obligate pathogen depends on the efficiency of direct host-to-host transmission, and consequently, too high or too low levels of virulence could be detrimental from the parasites perspective [2, 3]. If host death via high virulence does not reduce the chances of getting transmitted, increase in virulence is often expected because it is thought to be an inevitable consequence of within-host adaptation and increased parasite reproduction [2, 4, 5]. Opportunistic pathogens are at least partially disconnected from the transmission-virulence feedback loop because, by definition, they can survive and proliferate independently from the host in the environment [6–8]. Questions remain, which selective forces drive the evolution of high virulence in environmentally growing opportunists, and on the other hand, what factors may limit the virulence evolution [9]? Transition from strictly environmental strategy towards pathogenesis can evolve by “coincidental” selection in the outside-host environment because many functional virulence traits might be beneficial for free-living environmental bacteria [10–12]. For example, selection by protozoan predators could indirectly help some bacterial pathogens to acquire the tools for establishing a novel niche within the host [13]. However, protozoan grazing can also lead to an opposite evolutionary outcome: a trade-off between anti-predatory adaptation and virulence [14–16]. Barret et al. [17] and Vial et al. [18] have shown that intraspecies competition, both in the free-living environment and within hosts, can maintain polymorphism in virulence. Thus, the ecological interplay between outside-host and within-host environments drives the life-history evolution in opportunistic pathogens [8, 9]. The relative significance of within-host and outside-host selection could however differ massively between free-living pathogen species and even between strains of the same species.

Trade-offs between environmental and within-host strategies in *S. marcescens* and other opportunists have been found [15, 19–21], leading to a situation where pathogen’s outside-host fitness improves at the cost of decreased virulence. It is unclear, however, what direction evolution of virulence takes if selection occurs primarily within the host. Letting an obligate pathogen continuously infect hosts without paying any transmission costs typically leads to a rapid increase in virulence, which is considered as strong evidence for a transmission vs. virulence trade-off (reviewed in [4]). The trade-off is also indirectly supported by the tendency of environmentally persistent pathogens to be more virulent than less persistent ones. In other words, persistence to outside-host environment could make pathogens less dependent on the host-to-host contact (direct transmission) consequently allowing higher virulence [22, 23]. It has to be noted, however, that in phage-bacterium systems an opposite relationship between persistence and virulence has been found [24, 25].

Just like the “conventional wisdom” view [26, 27] of pathogens inevitably evolving towards mutualism, also the view of maximum virulence being the most efficient strategy to draw resources from the host, seems overly simplified [10, 28, 29]. We propose that within-host selection might as well lead to fitness increase without increased virulence. In other words, high virulence could be selected against within the host. For example, the notorious enterohemorrhagic *Eschericia coli* does not necessarily gain fitness benefit from the disease causing shiga-toxin production in human hosts [30]. Thus, within-host environment has potential to select against virulence traits even without epidemiological costs of lowered transmission. This would be even more feasible expectation if virulence factors were costly [19, 31, 32]. Virulence could also evolve rapidly towards a non-optimal direction, up or down, during a host change or when a new genotype emerges in a pathogen population [33]. It is also possible that adaptation to fluctuating selection pressures, between hosts and within the hosts, is costly and leads to reduced virulence in comparison to selection only in the outside-host or within-host environment [34].

We tested experimentally how the virulence of an opportunistic bacterial pathogen *S. marcescens* evolves due to within-host selection. We used a serial passage setup, where strains descending from a common ancestor were sequentially transferred from host to host in *Drosophila melanogaster* with a natural transmission route through contaminated food. We measured how selection during the infections changed bacterial life-history traits including growth rate, biomass yield, swarming motility, extracellular protease activity and virulence. We then compared the replicated selection lines to lines that evolved in an artificial outside-host environment (to control for the effect of growth medium that was used for oral infection), and to the ancestral bacteria. Reduced virulence in the within-host treatment suggests that pathogens can evolve towards a more benign state because of selection pressures acting exclusively inside the host, or because of rapid fluctuations between within-host and outside-host selection.

## Materials and methods

### Study species

*Serratia marcescens* is a gram-negative, cosmopolitan enterobacterium that inhabits a large variety of environments [35]. It is an opportunistic pathogen being able to infect an extremely vast range of hosts, for example plants, nematodes, insects, fish, and mammals via environmental transmission [35–37]. The *S. marcescens* db11 strain was originally isolated from a moribund fruit fly [38] and thus, has some degree of pre-adaptation to the within-host environment used in the experiment.

*Drosophila melanogaster* wild type Oregon R strain was used as a model host. They were reared on semolina-sugar-yeast-agar medium in 25 °C and population size was kept >1000 at all times.

### Evolutionary treatments

A single ancestral clone of *S. marcescens* db11 [38] was isolated from a frozen stock and divided into the two evolutionary treatments with ten replicate strains each.

The within-host treatment was serial passaged through *Drosophila melanogaster* as follows: The cryopreserved (500 μl bacteria + 500 μl 80 % glycerol in −80 °C) replicate strains were thawed and grown in 2 ml of Luria-Bertani (LB) growth medium (10 g peptone, 5 g yeast extract, 10 g NaCl in 1 L of dH_2_O) overnight after which 800 μl of the solution was mixed 1:1 with 50 mM sucrose solution. The 1.6 ml sugar-bacteria mix was then soaked into a dentist’s cotton roll (Top Dent, Lifco Dental AB, Enköping, Sweden), which was folded on a bottom of a 23 ml *Drosophila*–culture vial (Sarstedt AG and Co, Nümbrecht, Germany). Ten flies (anesthetised with CO_2_) were added to the vial, which was then sealed with a cotton plug. The flies were let to feed on the solution and after 65 h they were picked into 1.5 ml Eppendorf tubes. On the occasions that there were still live flies in the tubes, they were first anesthetised. The flies were then surface sterilized: they were rinsed with 1 ml of 1.) dH_2_O, 2.) 70 % EtOH, 3.) dH_2_O, 4.) 5 % sodium hypochlorite and 5.) 3 × dH_2_O. The tubes were rigorously vortexed between every step. The method works reliably according to a preliminary experiment where healthy flies were first dipped in high-density bacterial solution, surface sterilized, and tested negative for Serratia (data not shown). This way, it was also confirmed that our stock flies did not already have a chronic *S. marcescens* infection. The flies were then homogenized, homogenates diluted 1:2000 in sterile water, and 100 μl of the solution cultivated on *S. marcescens* –selective agar plate (42 g deoxyribonuclease test agar with methyl green, 10 g L-arabinose, 5 mg phenol red, 4 ml 1 % methyl green, 10 mg ampicillin, 10 mg colistimethate, 20 mg cephalothin, and 5 mg amphotericin B in 1 l of dH_2_O, medium modified from [35]). Plates were grown overnight and bacterial mass was collected with a sterile loop and cryopreserved. For a next passage, 5 μl of the cryopreserved solution was grown overnight in 2 ml LB, after which it was ready to be mixed with sucrose and to infect the next cycle of flies.

The outside-host control treatment went through an identical environment than the within-host treatment, essentially excluding the flies: 800 μl of bacteria were mixed 1:1 with sucrose in a fly vial with the dental roll for 65 h, after which the roll was soaked in 2 ml of sterile water, sampled 10 μl with a pipette, diluted 1:2000, cultivated on the selective plates and finally cryopreserved for the next passage.

The passages were repeated ten cycles and the strains were cryopreserved as described before. Ten random clones per replicate strain were then isolated by dilution plating, resulting in 200 clones and the ancestor. Cryopreserved clone libraries were created on four 100-well spectrophotometer plates (Growth Curves Ltd., Helsinki, Finland) in randomized order as described in [20], with 20 wells holding replicate clones of the ancestor.

### Life-history trait measurements

#### Growth parameters

For the growth trait measurements, the clone libraries were replicated on spectrophotometer plates containing fresh medium. Maximum growth rate and maximum population size were then measured in LB-sucrose medium (the same medium that was used in the evolutionary treatments) with Bioscreen C™ spectrophotometer (Growth Curves Ltd, Helsinki, Finland). Optical density (OD) was measured at 600 nm, 5 min interval, 25 °C, 400 μl volume. From the log transformed raw data, a MATLAB script was used to find a maximum linear slope in a 30 time points (2.5 h) sliding window to determine maximum growth rate for a clone. Similarly, the biomass yield was determined as a maximum mean of non-transformed OD in a 30 time points sliding window.

#### Motility and protease activity assays

Clones from the 10^th^ passage were grown overnight in 400 μl of LB-medium. Sterile 2 μl loop (VWR, Radnor, PA, USA) was dipped in the culture and then in the centre of a semi-solid motility agar plate (LB medium with 0.7 % agar). The plates were photographed after 55 h and the colonized area determined with ImagePro software [15]. The protease activity was measured on a 1 % skimmed milk agar plate as a diameter of the casein degradation halo after 48 h in 31 °C [39].

#### Virulence measurement

The flies (N = 2543) were infected with the individual clones in the same manner as was done with the strains in the evolutionary treatment and the mortality was recorded at three-hour intervals (the odd number of flies stems from rare cases where some individual flies did not for example wake up from anaesthesia and were excluded from the analysis). Thus, ca. 10 replicate flies were infected with 10 different clones from 10 replicate populations from the evolutionary treatments. 200 flies were infected with the ancestor. Flies fed with sterile sucrose solution were used as negative controls.

#### Bacterial load measurement

The bacteria were inoculated and fed to the flies in the same manner as in the within-host evolutionary treatment, except only one fly was used per one clone. The bacterial load measurement was carried out for half of the clones produced in the evolutionary experiment (N = 94). After 60 h the flies were surface sterilized as described earlier and finally homogenized in 200 μl dH_2_O. After vortexing and brief centrifugation, 100 μl subsamples of the homogenates were serially diluted in dH_2_O and cultivated on *S. marcescens* selective medium. Colony forming units (CFU) were counted from 1000 and 10 000 fold dilutions and used as relative bacterial load in the hosts.

### Statistical analyses

Linear mixed model with REML was used to analyse the changes in maximum growth rate (log-transformed), biomass yield, motility (log-transformed), protease activity, and bacterial load in the hosts between the evolutionary treatments (within-host and outside-host). Replicate population identity was nested as a random factor within the treatment in the model. In the ancestor comparisons, different treatment groups had unequal sample sizes, which might lead to inflated F-statistics. To avoid over interpretation of the results we analysed the comparisons against the ancestral bacteria with Kruskall-Wallis rank test that is not sensitive to unequal sample sizes and different distributions: The pairwise differences in the population means of both evolutionary treatments between the ancestor were Bonferroni-corrected. Virulence of the clones was analyzed with Cox regression using treatment and replicate population identity as categorical covariates. The analyses were performed using SPSS statistics 21.0 (IBM).

## Results

### Growth traits

**Fig. 1:**
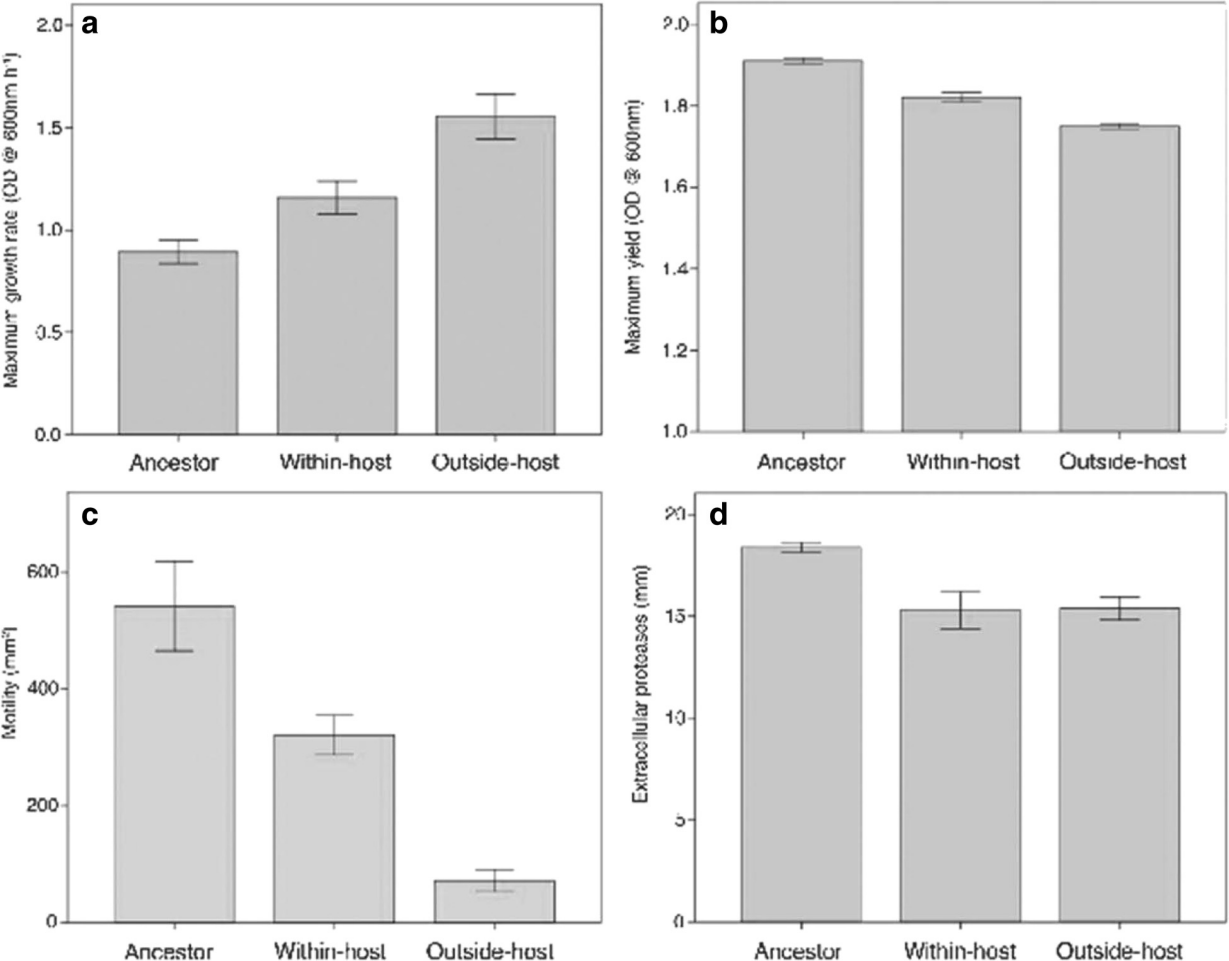
Evolved bacterial life-history traits. Maximum growth rate **a**, Maximum biomass yield **b**), Motility **c**), and secreted extracellular proteases **d**). The evolutionary treatment (ancestor, within-host, and outside-host) is on the x-axis. Error bars denote +/− SE

The treatment had a significant effect on maximum growth rate (H = 13.8, df = 2 *p* = 0.001). The ancestor (A) had lowest mean growth rate, followed by the within-host (WH) treatment, and the highest growth rate evolved in the outside-host (OH) treatment (A vs. WH: H = 5.6, *p* = 0.648; A vs. OH: H = 16.8, *p* = 0.001; WH vs. OH: F_1, 184_ = 12.1, *p* = 0.001). With maximum biomass yield (H = 19.7, df = 2 *p* < 0.001), the trend was the opposite (A vs. WH: H = 11.4, *p* = 0.035; A vs. OH: H = 19.4, *p* < 0.001; WH vs. OH: F_1, 119_ = 3.1, *p* = 0.08). Treatment means are presented in Figure 1a and b.

### Motility

**Fig. 2:**
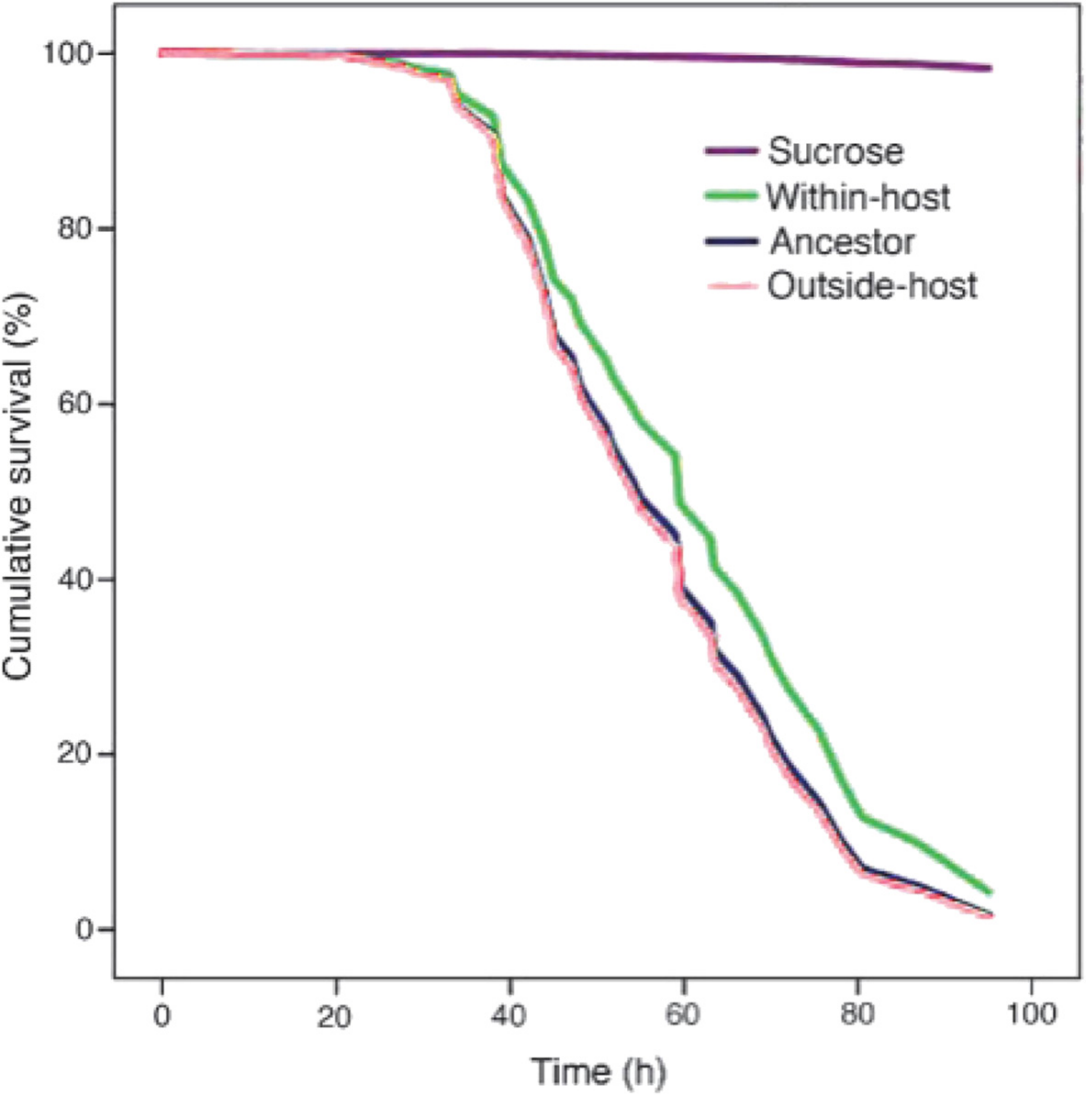
*D. melanogaster* mortality when infected with the two treatments of evolved bacteria or the ancestor. Sucrose solution was used for negative controls

The treatment (H = 24.5, df = 2, *p* < 0.001) had a significant effect on swarming motility of the clones: The ancestor was most motile, within-host treatment and outside-host treatment had lost some of their swarming ability (Fig. 2a, A vs. WH: H = 10.0, *p* = 0.084; A vs. OH: H = 22.3, *p* < 0.001; WH vs. OH: F_1, 87_ = 0.005, *p* = 0.928).

### Protease activity

Both treatments (H = 16.5, df = 2, *p* < 0.001) decreased protease activity compared to the ancestor (Fig. 2b, A vs. WH: H = 14.0, *p* = 0.005; A vs. OH: H = 15.6, *p* = 0.001; WH vs. OH: F_1, 87_ = 3.3, *p* = 0.07).

### Virulence

The death rate of the flies differed between the evolutionary treatments (*N* = 2543, df = 3, Wald = 148.4, *p* < 0.001). The ancestor and outside-host treatment were indistinguishable while the within-host treatment had attenuated virulence. The negative controls, i.e. flies fed with sterile sugar solution, had almost zero mortality (Table 1, Fig. 2).

**Table 1:** Pairwise differences in virulence between the evolved bacteria (within-host and outside-host treatments), ancestor, and negative controls (sucrose) in the infection experiment

	*sucrose*	*ancestor*	*within-host*	*outside-host*
*sucrose*		*Wald = 136.700* *SE = 0.457* Exp(B) = 241.359 p < 0.001*	*Wald = 120.118* *SE = 0.463* Exp(B) = 186.303 p < 0.001*	*Wald = 135.346* *SE = 0.462* Exp(B) = 251.699 p < 0.001*
*ancestor*			*Wald = 4.248* *Exp(B) = 0.772* SE = 0.129 p = 0.039*	*Wald = 0.087* *Exp(B) = 1.043* *SE = 0.127 p = 0.768*
*within-host*				*Wald = 4.251* *Exp(B) = 1.351* *SE = 0.147* p = 0.039*

### Bacterial load

**Fig. 3:**
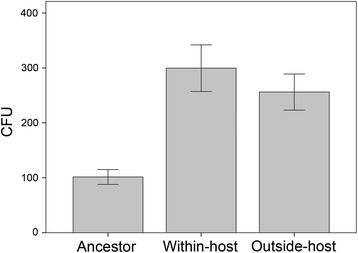
Relative bacterial load (CFU) in the hosts 60 h after infection. The evolutionary treatment (ancestor, within-host, and outside-host) is on the x-axis. Error bars denote +/− SE

The evolutionary treatment did not affect the bacterial load in the host (Kruskall-Wallis with ancestor included: H = 5.4, df = 2, *p* = 0.07; Linear mixed model between the evolutionary treatments: F_1, 84_ = 0.7, *p* = 0.42, Fig. 3).

## Discussion

A vast amount of theoretical and empirical evidence suggests that high pathogen virulence evolves within the host if it is not restricted by lowered probability of transmission between the hosts [2, 4]. Contrary to this, we show that within-host selection attenuates virulence in an opportunistic pathogen *Serratia marcescens* even when the chances of getting transmitted to the next host were standardised by manipulation.

*In vivo* serial passages often lead to evolution of high virulence, apparently due to relaxed transmission costs [4]. Here we found selection to act against high virulence in conditions with a very high transmission probability, which, in the light of the traditional trade-off model for evolution of virulence is a counterintuitive result [3, 29]. However, in spite of the scarce experimental evidence, implications that lowered virulence can be an adaptation to within-host environment do exist. For example, some viruses have evolved traits that prevent host damage in order to avert the clearance by the immune system [10, 40]. *Pseudomonas aeruginosa* loses some of its virulence factors in response to within-host adaptation when the acute infection becomes chronic. This happens because the production of virulence factors is costly and leads to a competitive disadvantage after the host colonisation has been established [21, 31]. Similar phenomenon, coined as strain replacement, has been suggested to take place in progressive HIV infection in humans [41]. Within-host evolution of virulence could also be due to co-operation, cheating, or spite occurring because of changes in the relatedness of infection [32].

In this particular *Drosophila*-*Serratia* system the dose used for infection may have been higher than in natural circumstances. This could partially relax the selection for effective and early colonisation of the host that can lead to high virulence. Therefore, virulence would deteriorate if within-host competition in concert with the inhibition by the host’s immune system selected less harmful clones passage after passage. Colonisation-virulence trade-off could nevertheless occur: If the clones that infect the host the fastest were the least virulent, they might be able to block the invasion of the most virulent genotypes through direct strain competition. This would be an opposite scenario compared to the example with *P. aeruginosa* where the most virulent clones establish the infection and are gradually replaced by the more benign genotypes [31].

Extracellular protease production, one of the most potential candidates as a virulence factor because it can cause gut penetration [42–44], was not responsible for the change in host mortality in this system. Virulence factors can be energetically costly to produce [45] and they can act as immune elicitors that evoke host defence [28, 29]. Thus, selection against some of such traits could lead to better growth in the gut environment but at the same time to reduced ability to penetrate the gut epithelium, consequently lowering virulence but increasing pathogen fitness. Similarly to proteases, motility that is also directly connected to virulence in several bacterial species [46, 47] did not evolve within the host, but instead the change from the ancestor was apparently an adaptation to the growth medium. This conclusion stems from most of the measured traits evolving towards the same direction as the outside-host treatment (Fig. 1) that acted as a control for the evolutionary change due to the experimental infection process. It has to be noted, however, that using the oral infection with contaminated food always requires some kind of medium, and that this situation most likely resembles the natural route for host entrance in environmental opportunists. For the same reason it was impossible to control the exact bacterial dose that the larvae ingested in this setting. Thus it could be argued that the changed growth traits might be responsible for the differences in virulence through a dose effect. However, the fact that the within-host treatment with attenuated virulence was intermediate in both of the growth traits strongly suggests that this was not the case. The bacterial loads in the hosts 60 h after the infection did not differ between the evolutionary treatments, which further supports the conclusion that virulence really attenuated without a dose effect. Although we could not identify the traits behind high virulence, our results clearly imply that a pathogen can evolve towards a more benign state if virulence factors are not directly selected for, or if hiding from the host immune system requires attenuated virulence [31].

While the medium itself did not select against virulence, it is possible that the rapid fluctuation between the two very different environments, within and outside the host, did. This could be seen as an analogous situation with an obligate pathogen alternating with two different hosts, or between a vector and a host. For example, it has been shown that continuous switching between host types can constrain the evolution of a pathogen in both hosts [47]. In a natural setting where outside-host environment is constantly changing, the trade-offs between the non-host and within-host situations could be even bigger. Indeed, evolution of generalism through fluctuating temperature in liquid culture can trade off with virulence in *S. marcescens* [20]. Previous work with an environmental isolate of *S. marcescens* also suggests that virulence traits might deteriorate outside the host in some replicate populations even without direct negative selection [15, 20]. In this study, however, the outside-host evolved bacteria maintained their virulence at the same level with the ancestor that was originally isolated from a fruit fly. Compared to the previous studies, we used a different *S. marcescens* strain, and the length of this experiment was shorter. It is possible that longer experiment would have caused potentially unused traits to disappear from the population because of drift or relaxed selection [15]. In environmentally transmitted opportunistic pathogens, conflicting selection pressures between the outside-host and within-host conditions are likely to be part of the natural lifecycle [8, 9, 15]. The switching between environmental reservoir and the host may have led to more genetically diverse populations of bacteria. Attenuated virulence could then be caused by diversity-induced decrease in co-operation: Population level increase in the frequency of cheating genotypes that do not produce public goods virulence factors can cause lower host mortality especially when the realized infections are clonal [48–50].

## Conclusions

This study demonstrates that within-host evolution can lead to lower parasite virulence, even when the epidemiological cost of reduced transmission is removed. The full understanding of the evolution of lowered virulence in this system certainly requires further work to provide a mechanistic explanation. Nevertheless, our results challenge the traditional trade-off model for the evolution of virulence in capturing the full variety of selection pressures operating in host-parasite interaction [3, 6, 8, 29]. Instead of exclusively trading virulence off with host-to-host transmission, pathogens could also improve their performance within the hosts by being more benign, or they might pay an evolutionary cost of adapting to fluctuations between within-host and non-host situations. Especially, this should be taken into account in the management of environmentally transmitted opportunists, which do not conform to the same epidemiological framework with obligatory pathogens but still cause severe threat to human health.
